# Melatonin suppresses senescence‐derived mitochondrial dysfunction in mesenchymal stem cells via the HSPA1L–mitophagy pathway

**DOI:** 10.1111/acel.13111

**Published:** 2020-01-22

**Authors:** Jun Hee Lee, Yeo Min Yoon, Keon‐Hyoung Song, Hyunjin Noh, Sang Hun Lee

**Affiliations:** ^1^ Medical Science Research Institute Soonchunhyang University Seoul Hospital Seoul Korea; ^2^ Departments of Biochemistry Soonchunhyang University College of Medicine Cheonan Korea; ^3^ Department of Pharmaceutical Engineering College of Medical Science Soonchunhyang University Asan Korea; ^4^ Department of Internal Medicine Soonchunhyang University Seoul Korea; ^5^ Hyonam Kidney Laboratory Soonchunhyang University Seoul Korea

**Keywords:** HSPA1L, melatonin, mesenchymal stem cells, mitochondria, mitophagy, replicative senescence

## Abstract

Mesenchymal stem cells (MSCs) are a popular cell source for stem cell‐based therapy. However, continuous ex vivo expansion to acquire large amounts of MSCs for clinical study induces replicative senescence, causing decreased therapeutic efficacy in MSCs. To address this issue, we investigated the effect of melatonin on replicative senescence in MSCs. In senescent MSCs (late passage), replicative senescence decreased mitophagy by inhibiting mitofission, resulting in the augmentation of mitochondrial dysfunction. Treatment with melatonin rescued replicative senescence by enhancing mitophagy and mitochondrial function through upregulation of heat shock 70 kDa protein 1L (HSPA1L). More specifically, we found that melatonin‐induced HSPA1L binds to cellular prion protein (PrP^C^), resulting in the recruitment of PrP^C^ into the mitochondria. The HSPA1L‐PrP^C^ complex then binds to COX4IA, which is a mitochondrial complex IV protein, leading to an increase in mitochondrial membrane potential and anti‐oxidant enzyme activity. These protective effects were blocked by knockdown of HSPA1L. In a murine hindlimb ischemia model, melatonin‐treated senescent MSCs enhanced functional recovery by increasing blood flow perfusion, limb salvage, and neovascularization. This study, for the first time, suggests that melatonin protects MSCs against replicative senescence during ex vivo expansion for clinical application via mitochondrial quality control.

## Graphical abstract

Replicative cellular senescence in MSCs increases mitofusion of damaged mitochondria, leading to suppression of mitophagy. Melatonin increases the expression of HSPA1L, leading to the recruitment of PrPC, which induces mitofission of damaged mitochondria, resulting in the increase in the mitochondrial activities. Melatonin‐treated senescent MSCs improve survival, proliferation, and neovascularization in ischemic disease.

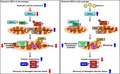

## 1 INTRODUCTION

Human mesenchymal stem cells (MSCs) are powerful cell therapy tools for clinical trials in ischemic diseases, due to their self‐renewal and multiple differentiation potential (Si, Zhao, Hao, Fu, & Han, 2011). Over 500 clinical trials assessing MSC‐based therapy in several diseases (http://www.clinicaltrials.gov) have shown that it may be possible to use MSCs for therapeutic use due to the acquisition of a large number of clinical‐grade MSCs from adipose tissues and bone marrow by ex vivo expansion (Squillaro, Peluso, & Galderisi, 2016; Uccelli, Moretta, & Pistoia, 2008). Although inter‐individual variability in MSCs isolated from donors is excluded, serial cell expansion induces replicative senescence, which can affect the clinical‐grade MSC safety and the clinical therapeutic efficacy of MSCs (Hayflick, 1965; Loisel et al., 2017; Tarte et al., 2010). Therefore, the investigation on the replicative senescence of human MSCs for clinical application and its molecular mechanism is important for enhancing the clinical efficacy of MSCs.

Replicative senescence is associated with the inhibition of the regenerative potential of stem and progenitor cells (Correia‐Melo & Passos, 2015). In particular, senescent cells increase the generation of reactive oxygen species (ROS), resulting in mitochondrial dysfunction (Korolchuk, Miwa, Carroll, & von Zglinicki, 2017). In healthy cells, mitochondrial homeostasis is well‐regulated by mitochondrial quality control mechanisms, mitochondrial fission/fusion, and mitophagy. Replicative senescence impairs mitochondrial quality control, resulting in decreased mitochondrial respiratory coupling, increased ROS production, and, ultimately, dysfunctional mitochondria (Korolchuk et al., 2017; Ni, Williams, & Ding, 2015). However, it is currently unclear how replicative senescence dysregulates mitofission/fusion and mitophagy processes in mitochondrial quality control.

Melatonin is associated with several physiological functions, including sleep, circadian rhythms, and neuroendocrine actions (Jan, Reiter, Wasdell, & Bax, 2009). Accumulating evidence has also shown that melatonin regulates apoptosis, autophagy, endoplasmic reticulum stress, and anti‐oxidant effects (Fernandez, Ordonez, Reiter, Gonzalez‐Gallego, & Mauriz, 2015; Garcia et al., 2014; Reiter et al., 2016). Furthermore, preclinical studies suggest that melatonin enhances the therapeutic potential of MSCs in myocardial infarction, chronic kidney disease (CKD), and hindlimb ischemia (Han et al., 2019; Lee, Han, & Lee, 2017; Lee, Jung, Oh, Yun, & Han, 2014). Our previous studies indicate that melatonin increases the regenerative potential of MSCs in ischemic disease and CKD through upregulation of cellular prion protein (PrP^C^) which is involved in self‐renewal, differentiation, and angiogenesis in stem and/or progenitor cells (Doeppner et al., 2015; Han et al., 2019; Lee, Han, & Lee, 2017). However, the mechanism by which melatonin‐induced PrP^C^ regulates the bioactivity of MSCs and protects them from stress and pathophysiological condition is still unclear. This study focused on the effect of melatonin on replicative senescence in MSCs through mitophagy. We also investigated the mechanisms by which melatonin‐induced mitophagy rescues replicative senescence‐associated dysfunction of mitochondria through regulation of heat shock protein, especially heat shock 70 kDa protein 1L (HSPA1L) by promoting the recruitment of PrP^C^ into mitochondria. Finally, we assessed the functional recovery in a murine hindlimb ischemia model by engrafting melatonin‐treated senescent MSCs which were cultured at late passage.

## 2 RESULTS

### 2.1 Replicative senescence increases damaged mitochondria in MSCs via impaired mitochondrial quality control

**Figure 1:**
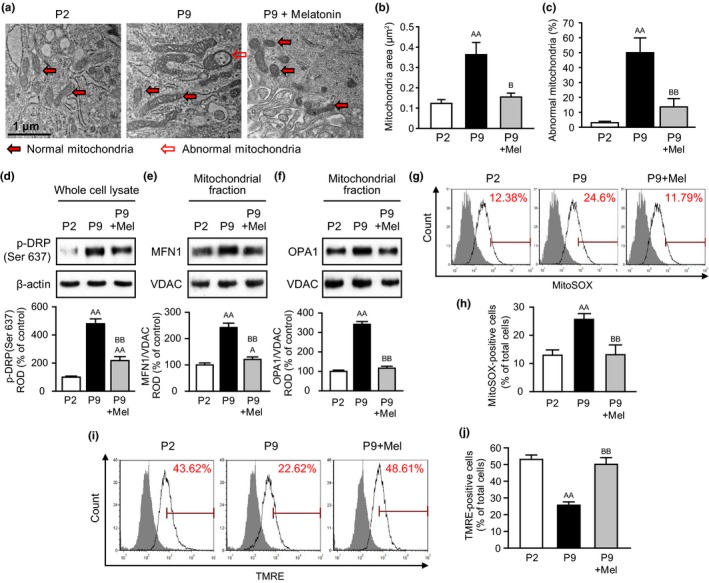
Replicative senescence induces abnormal mitochondria through defective control of mitochondrial quality. (a) Representative TEM images of healthy MSCs (passage 2; P2), senescent MSCs (passage 9; P9), and senescent MSCs treated with melatonin (1 μM; P9 + melatonin) for 24 hr. Scale bar = 1 μm. (b) Quantitative analysis of morphometric data from TEM images in healthy MSCs (P2), senescent MSCs (P9), and senescent MSCs treated with melatonin (P9 + Mel). Values represent the mean ± *SEM* (*n* = 10). ^AA^ *p* < .01 vs. P2 and ^B^ *p* < .05 vs. P9. (c) Percentages of abnormal mitochondria which were swollen with evidence of severely disrupted cristae throughout a mitochondrion obtained from TEM images in healthy MSCs (P2), senescent MSCs (P9), and senescent MSCs treated with melatonin (P9 + Mel). Values represent the mean ± *SEM* (*n* = 5). ^AA^ *p* < .01 vs. P2 and ^BB^ *p* < .01 vs. P9. (d–f) Expression of p‐DRP (Ser 637) (d), MFN1 (e), and OPA1 (f) in healthy MSCs (P2), senescent MSCs (P9), and senescent MSCs treated with melatonin (P9 + Mel). The levels of p‐DRP (Ser 637) (d), MFN1 (e), and OPA1 (f) were determined by densitometry relative of β‐actin and VDAC, respectively. Values represent the mean ± *SEM* (*n* = 3). ^A^ *p* < .05; ^AA^ *p* < .01 vs. P2 and ^BB^ *p* < .01 vs. P9. (g–j) Flow cytometry for MitoSOX (g and i) and TMRE (i and j) in healthy MSCs (P2), senescent MSCs (P9), and senescent MSCs treated with melatonin (P9 + Mel). Values represent the mean ± *SEM* (*n* = 5). ^AA^ *p* < .01 vs. P2 and ^BB^ *p* < .01 vs. P9

To investigate whether replicative senescence induces abnormal mitochondria in MSCs, we assessed the morphological change of mitochondria in MSCs at early (P2) and late passage (P9) by transmission electron microscopy (TEM) analysis (Figure 1a). Morphological analysis showed that replicative senescence in MSCs significantly increases abnormal mitochondria (Figure 1b,c). To examine the effect of melatonin on mitochondria in senescent MSCs, we analyzed the morphology, quality control, and function of mitochondria in MSCs at P9. Melatonin significantly inhibited replicative senescence‐induced abnormal mitochondrial area and size (Figure S1, Figure 1a–c). Western blot analysis for mitofusion‐associated proteins, such as phosphor‐dynamin‐1‐like protein at Ser 637 (p‐DRP (Ser 637)) as the inactive state of a mitochondrial fission‐associated protein (Kashatus et al., 2011; Wang et al., 2012), mitofusion‐1 (MFN1), and optic atrophy 1 (OPA1), suggested that replicative senescence significantly increased the expression of mitofusion‐associated proteins, whereas melatonin significantly decreased the level of mitofusion‐associated proteins in senescent MSCs (Figure 1d–f). Furthermore, mitofission‐associated protein, total DRP1 was significantly augmented in senescent MSCs treated with melatonin (Figure S2). In addition, replicative senescence significantly increased the generation of ROS and decreased mitochondrial membrane potential in MSCs, whereas melatonin significantly inhibited the production of ROS and increased mitochondrial membrane potential in senescent MSCs (Figure 1g–j). These results indicate that melatonin protects mitochondrial dysfunction in MSCs from replicative senescence through inhibition of mitofusion.

### 2.2 Melatonin facilitates the recruitment of PrP^C^ into mitochondria through upregulation of HSPA1L

**Figure 2:**
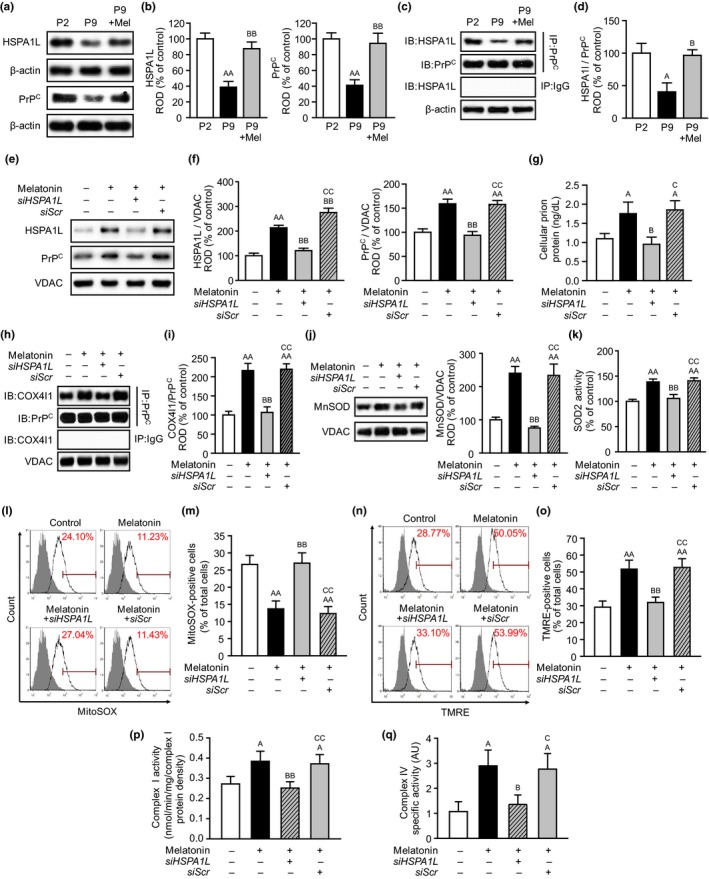
Melatonin‐induced HSPA1L facilitates the recruitment of PrP^C^ in mitochondria in senescent MSCs through binding of PrP^C^ with COX4I1. (a) Expression of HSPA1L and PrP^C^ in healthy MSCs (P2), senescent MSCs (P9), and senescent MSCs treated with melatonin (P9 + Mel). (b) The levels of HSPA1L and PrP^C^ were determined by densitometry relative of β‐actin, respectively. Values represent the mean ± *SEM* (*n* = 3). ^AA^ *p* < .01 vs. P2 and ^BB^ *p* < .01 vs. P9. (c) Co‐immunoprecipitation of HSPA1L with PrP^C^ in healthy MSCs (P2), senescent MSCs (P9), and senescent MSCs treated with melatonin (P9 + Mel). (d) The level of HSPA1L, whose binding with PrP^C^ was normalized to that of PrP^C^. Values represent the mean ± *SEM* (*n* = 3). ^A^ *p* < .05 vs. P2 and ^B^ *p* < .05 vs. P9. (e) Expression of HSPA1L and PrP^C^ in the mitochondrial fraction of senescent MSCs (passage 9). (f) The levels of HSPA1L and PrP^C^ were determined by densitometry relative of VDAC. Values represent the mean ± *SEM* (*n* = 3). ^AA^ *p* < .01 vs. nontreated senescent MSCs, ^BB^ *p* < .01 vs. melatonin‐treated senescent MSCs, and ^CC^ *p* < .01 vs. melatonin‐treated senescent MSCs pretreated with *HSPA1L* siRNA (*siHSPA1L*). (g) Concentration of PrP^C^ in senescent MSCs measured by ELISA. Values represent the mean ± *SEM* (*n* = 5). ^A^ *p* < .05 vs. nontreated senescent MSCs, ^B^ *p* < .05 vs. melatonin‐treated senescent MSCs, and ^C^ *p* < 0.05 vs. melatonin‐treated senescent MSCs pretreated with *siHSPA1L*. (h) Co‐immunoprecipitation of COX4I1 with PrP^C^ in senescent MSCs (P9). (i) The level of COX4I1, whose binding with PrP^C^ was normalized to that of PrP^C^ level. Values represent the mean ± *SEM* (*n* = 3). ^AA^ *p* < .01 vs. nontreated senescent MSCs, ^BB^ *p* < .01 vs. melatonin‐treated senescent MSCs, and ^CC^ *p* < 0.01 vs. melatonin‐treated senescent MSCs pretreated with *siHSPA1L*. (j) Expression of MnSOD in mitochondrial fraction of senescent MSCs. The level of MnSOD was determined by densitometry relative of VDAC. Values represent the mean ± *SEM* (*n* = 3). ^AA^ *p* < .01 vs. nontreated senescent MSCs, ^BB^ *p* < .01 vs. melatonin‐treated senescent MSCs, and ^CC^ *p* < .01 vs. melatonin‐treated senescent MSCs pretreated with *siHSPA1L*. (k) The activity of SOD2 in senescent MSCs. Values represent the mean ± *SEM* (*n* = 3). ^AA^ *p* < .01 vs. nontreated senescent MSCs, ^BB^ *p* < .01 vs. melatonin‐treated senescent MSCs, and ^CC^ *p* < .01 vs. melatonin‐treated senescent MSCs pretreated with *siHSPA1L*. (l–o) Flow cytometry for MitoSOX (l and m) and TMRE (n and o) in senescent MSCs. Values represent the mean ± *SEM* (*n* = 5). ^AA^ *p* < .01 vs. nontreated senescent MSCs, ^BB^ *p* < .01 vs. melatonin‐treated senescent MSCs, and ^CC^ *p* < .01 vs. melatonin‐treated senescent MSCs pretreated with *siHSPA1L*. (p and q) The activity of mitochondrial complex I (p) and IV (q) in senescent MSCs. Values represent the mean ± *SEM* (*n* = 3). ^A^ *p* < .05 vs. nontreated senescent MSCs, ^B^ *p* < .05; ^BB^ *p* < .01 vs. melatonin‐treated senescent MSCs, and ^C^ *p* < .05; ^CC^ *p* < .01 vs. melatonin‐treated senescent MSCs pretreated with *siHSPA1L*

Previous studies have shown that PrP^C^ binds to PTEN‐induced kinase 1 (PINK1) which regulates mitochondrial quality control through the mitophagy process (Han et al., 2019; Yoon et al., 2019). To understand whether melatonin stabilizes the expression of PrP^C^ for recruitment into mitochondria in senescent MSCs via chaperone protein, we focused on HSPA1L, which is a member of the heat shock protein 70 (HSP70) family and contributes to stabilization of protein, signal transduction, and protein homeostasis (Mayer & Bukau, 2005). The expression of HSPA1L was significantly decreased in senescent MSCs (P9), compared to that in healthy MSCs (P2; Figure 2a,b). However, melatonin significantly increased the level of HSPA1L in senescent MSCs (Figure 2a,b). Interestingly, the PrP^C^ level in senescent MSCs was also significantly decreased, compared to that in healthy MSCs, whereas melatonin significantly augmented the expression of HSPA1L and PrP^C^ in senescent MSCs (Figure 2a,b). These findings suggested that the expression of PrP^C^ might be regulated by melatonin‐induced HSPA1L in senescent MSCs. To reveal the relationship between HSPA1L and PrP^C^ in MSCs, co‐immunoprecipitation of HSPA1L with PrP^C^ was performed in MSCs (Figure 2c). HSPA1L bound to PrP^C^ in MSCs, and this binding was significantly decreased by replicative senescence (Figure 2c,d). However, melatonin significantly facilitated the binding of HSPA1L with PrP^C^ in senescent MSCs (Figure 2c,d). To further investigate whether binding of HSPA1L with PrP^C^ induces the recruitment of PrP^C^ into mitochondria in senescent MSCs, the expression of HSPA1L and PrP^C^ in the mitochondria of senescent MSCs was assessed (Figure 2e). Melatonin significantly increased the localization of PrP^C^ into mitochondria in senescent MSCs through upregulation of HSPA1L, but knockdown of HSPA1L blocked this effect (Figure 2e,f). In addition, the level of PrP^C^ in senescent MSCs was significantly increased by treatment with melatonin, whereas silencing of HSPA1L decreased the expression of PrP^C^ (Figure 2g). These data indicate that melatonin increases the expression of HSPA1L and the binding of HSPA1L with PrP^C^, resulting in the stabilization of PrP^C^ and the recruitment of PrP^C^ into mitochondria.

### 2.3 Melatonin increases mitochondrial function via the HSPA1L‐PrP^C^‐COX4I1 complex

A previous study has revealed that the cytochrome *c* oxidase subunit 4 isoform 1 (COX4I1)‐HSP70 complex plays a pivotal role in the formation of cytochrome *c* oxidase (mitochondrial complex IV), leading to maintenance of mitochondrial membrane potential (Bottinger et al., 2013). To investigate whether PrP^C^ binds to COX4I1 and whether the melatonin‐mediated complex of PrP^C^‐COX4I1 regulates mitochondrial function in senescent MSCs through HSPA1L expression, we analyzed the interaction between PrP^C^ and COX4I1 by co‐immunoprecipitation. Co‐immunoprecipitation of COX4I1 with PrP^C^ indicated that COX4I1 bound to PrP^C^ in the mitochondria of senescent MSCs and that melatonin significantly increased the binding of COX4I1 with PrP^C^ (Figure 2h,i). However, this interaction was blocked by knockdown of HSPA1L, suggesting that the binding of COX4I with PrP^C^ is dependent on the melatonin‐mediated HSPA1L expression (Figure 2h,i). To further assess the effect of melatonin on mitochondrial function in senescent MSCs, we analyzed the generation of ROS and the mitochondrial membrane potential in senescent MSCs. The expression of manganese superoxide dismutase (MnSOD) and SOD2 activity was significantly increased in melatonin‐treated senescent MSCs, compared to that in nontreated senescent MSCs (Figure 2j,k). In addition, melatonin significantly decreased the production of ROS in senescent MSCs (Figure 2l,m). However, these melatonin effects were inhibited by silencing of HSPA1L (Figure 2j–m). Furthermore, melatonin significantly augmented the mitochondrial membrane potential in senescent MSCs through the increase in activities of mitochondrial complex I and IV, whereas silencing of HSPA1L blocked enhancement of melatonin‐induced mitochondrial membrane potential (Figure 2n–q). These findings indicate that melatonin enhances the mitochondrial function of MSCs against replicative cellular senescence through upregulation of HSPA1L.

### 2.4 Melatonin induces mitophagy in senescent MSCs via HSPA1L expression levels

**Figure 3:**
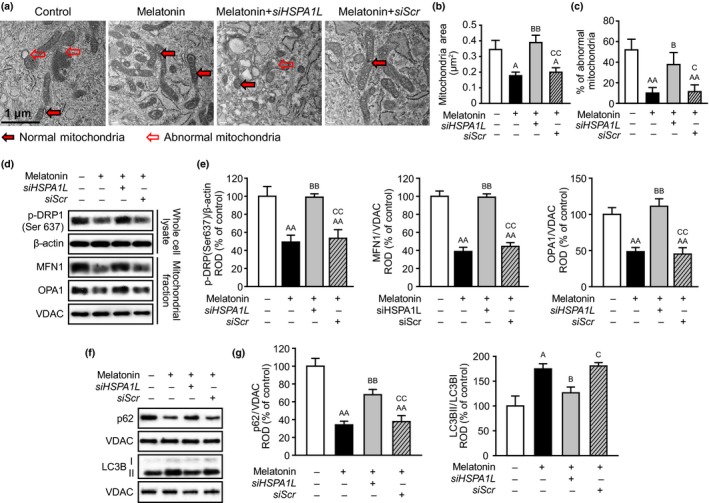
Melatonin induces mitophagy in senescent MSCs through expression of HSPA1L. (a) Representative TEM images of senescent MSCs (passage 9). Scale bar = 1 μm. (b) Quantitative analysis of morphometric data from TEM images in senescent MSCs. Values represent the mean ± *SEM* (*n* = 10). ^A^ *p* < .05 vs. nontreated senescent MSCs, ^BB^ *p* < .01 vs. melatonin‐treated senescent MSCs, and ^CC^ *p* < .01 vs. melatonin‐treated senescent MSCs pretreated with *siHSPA1L*. (c) Proportion of abnormal mitochondria which were swollen with evidence of severely disrupted cristae throughout a mitochondrion obtained from TEM images in senescent MSCs. Values represent the mean ± *SEM* (*n* = 5). ^AA^ *p* < .01 vs. nontreated senescent MSCs, ^B^ *p* < .05 vs. melatonin‐treated senescent MSCs, and ^C^ *p* < .05 vs. melatonin‐treated senescent MSCs pretreated with *siHSPA1L*. (d) Expression of p‐DRP (Ser 637), MFN1, and OPA1 in senescent MSCs. (e) The levels of p‐DRP (Ser 637), MFN1, and OPA1 were determined by densitometry relative of β‐actin and VDAC, respectively. Values represent the mean ± *SEM* (*n* = 3). ^AA^ *p* < .01 vs. nontreated senescent MSCs, ^BB^ *p* < .01 vs. melatonin‐treated senescent MSCs, and ^CC^ *p* < .01 vs. melatonin‐treated senescent MSCs pretreated with *siHSPA1L*. (f) Expression of p62 and LC3BII in mitochondrial fraction of senescent MSCs. (g) The levels of p62 and LC3BII/LC3BI were determined by densitometry relative of VDAC. Values represent the mean ± *SEM* (*n* = 3). ^A^ *p* < .05; ^AA^ *p* < .01 vs. nontreated senescent MSCs, ^B^ *p* < 0.05; ^BB^ *p* < 0.01 vs. melatonin‐treated senescent MSCs, and ^C^ *p* < .05; ^CC^ *p* < .01 vs. melatonin‐treated senescent MSCs pretreated with *siHSPA1L*

To explore the effect of melatonin on mitophagy in senescent MSCs through the expression of HSPA1L, we assessed the morphology of mitochondria in senescent MSCs after melatonin treatment. Treatment with melatonin significantly decreased the abnormal mitochondria, and knockdown of HSPA1L blocked the effect of melatonin on the removal of abnormal mitochondria in senescent MSCs (Figure 3a–c, Figure S3). The expression of mitofusion‐associated proteins, including p‐DRP1 (Ser 637), MFN1, and OPA1, was significantly decreased in melatonin‐treated senescent MSCs, compared to those in nontreated senescent MSCs (Figure 3d,e). Furthermore, decreased mitofusion processes enhanced mitophagy processes, such as the decrease in p62 expression and the increase in microtubule‐associated proteins 1A/1B light chain 3B (LC3BII) level (Figure 3f,g), resulting in the reduction of abnormal mitochondria in senescent MSCs (Figure 3a–c, Figure S3). Silencing of HSPA1L in melatonin‐treated senescent MSCs resulted in the significant increase in mitofusion and the significant suppression of mitophagy, resulting in the augmentation of abnormal mitochondria in melatonin‐treated senescent MSCs with knockdown of HSPA1L (Figure 3a–g, Figure S3). Mitofission‐associated protein, total DRP1, was also significantly inhibited in senescent MSCs treated with melatonin by knockdown of HSPA1L (Figure S4). Silencing of DRP1 showed the decrease in melatonin‐induced mitophagy in melatonin‐treated senescent MSCs (Figure S5). In addition, knockdown of MFN1 delayed replicative senescence in MSCs (Figure S6). Furthermore, melatonin significantly increased the levels of Parkin and PINK1 in mitochondria of senescent MSCs through upregulation of HSPA1L, indicating that this mitophagy‐mediated effect is distinguished from general autophagy (Figure S7). These results show that melatonin increases mitophagy in damaged mitochondria in senescent MSCs by regulating mitofusion through the expression of HSPA1L.

### 2.5 Melatonin rescues replicative cellular senescence of MSCs

**Figure 4:**
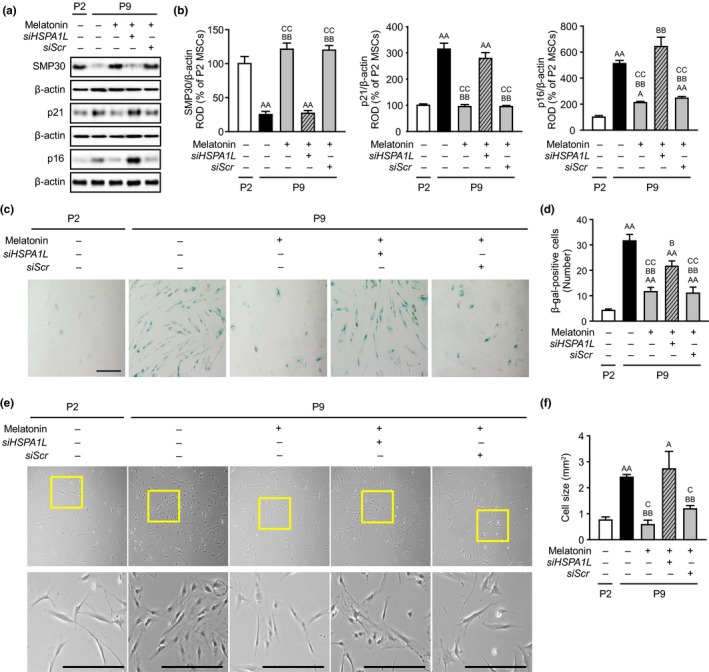
Melatonin‐induced HSPA1L protects replicative senescence in MSCs. (a) Expression of senescence marker proteins, including SMP30, p21, and p16 in healthy MSCs (P2) and senescent MSCs (P9). (b) The levels of SMP30, p21, and p16 were determined by densitometry relative of β‐actin, respectively. Values represent the mean ± *SEM* (*n* = 3). ^A^ *p* < .05; ^AA^ *p* < .01 vs. healthy MSCs (P2), ^BB^ *p* < .01 vs. senescent MSCs (P9), and ^CC^ *p* < .01 vs. melatonin‐treated senescent MSCs (P9) pretreated with *siHSPA1L*. (c) Senescence‐associated β‐galactosidase (SA‐β‐gal) assay in healthy MSCs (P2) and senescent MSCs (P9). Scale bar = 100 μm. (d) Number of SA‐β‐galactosidase‐positive cells. The values represent the means ± *SEM* (*n* = 3). ^AA^ *p* < .01 vs. healthy MSCs (P2), ^B^ *p* < .05; ^BB^ *p* < .01 vs. senescent MSCs (P9), and ^CC^ *p* < .01 vs. melatonin‐treated senescent MSCs (P9) pretreated with *siHSPA1L*. (e) Morphology of healthy MSCs (P2) and senescent MSCs (P9). Scale bar = 250 μm. (f) Quantification of cell size. The values represent the means ± *SEM* (*n* = 10). ^A^ *p* < .05; ^AA^ *p* < .01 vs. healthy MSCs (P2), ^BB^ *p* < .01 vs. senescent MSCs (P9), and ^C^ *p* < .05 vs. melatonin‐treated senescent MSCs (P9) pretreated with *siHSPA1L*

To examine whether melatonin protects MSCs against replicative senescence, senescence markers were assessed in healthy and senescent MSCs (Figure 4a). The expression of anti‐senescence marker senescence marker protein‐30 (SMP30) was significantly decreased in senescent MSCs (P9), compared to that in healthy MSCs (P2), and the levels of pro‐senescence markers, p21 and p16, were significantly increased in senescent MSCs (Figure 4a,b). In senescent MSCs, melatonin markedly increased the expression of SMP30 and decreased the levels of p21 and p16, but these effects were blocked by silencing of HSPA1L (Figure 4a,b). A senescence‐associated β‐galactosidase assay showed that melatonin significantly inhibited the cellular senescence in senescent MSCs through the expression of HSPA1L (Figure 4c,d). In addition, the morphological analysis in healthy and senescent MSCs indicated that melatonin reduced the enlarged cell size of senescent MSCs via HSPA1L level (Figure 4e,f). Furthermore, melatonin inhibited replicative senescence of MSCs isolated from different donor via HSPA1L expression (Figure S8). Melatonin also increased the expression of cell cycle‐associated proteins, including cyclin‐dependent kinase 2 (CDK2), cyclin E, CDK4, and cyclin D1, and the activation of survival and proliferation‐mediated signaling, such as protein kinase B (Akt), mammalian target of rapamycin (mTOR), and extracellular signal‐regulated kinase (ERK) in senescent MSCs (Figure S9). These data indicate that melatonin recovers replicative cellular senescence of MSCs through regulation of HSPA1L.

### 2.6 Melatonin‐treated senescent MSCs improve neovascularization in a hindlimb ischemia model

**Figure 5:**
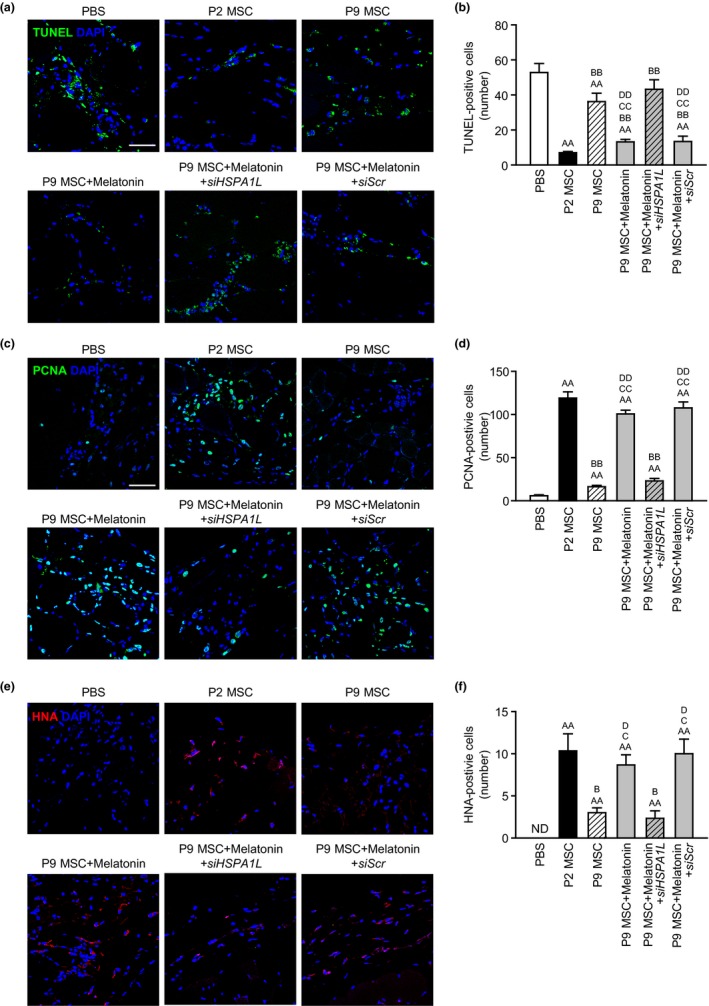
Anti‐apoptotic and proliferative effects of melatonin on transplanted senescent MSCs in ischemic injured tissues. At postoperative day 3, the ischemic injured sites of a murine hindlimb ischemia model were analyzed for the extent of apoptosis and proliferation of affected local cells after transplantation of senescent MSCs. (a) Apoptotic cells were analyzed by the TUNEL (green) assay. Scale bar = 50 μm. (b) The number of apoptotic cells was quantified by counting the number of TUNEL‐positive cells. The values represent the means ± *SEM* (*n* = 3). ^AA^ *p* < .01 vs. PBS, ^BB^ *p* < .01 vs. P2 MSC, ^CC^ *p* < .01 vs. P9 MSC, and ^DD^ *p* < .01 vs. P9MSC + melatonin+siHSPA1L. (c) Proliferating cells were analyzed by immunofluorescence staining for PCNA (green). Scale bar = 50 μm. (d) The number of proliferating cells was quantified by counting the number of PCNA‐positive cells. The values represent the means ± *SEM* (*n* = 3). ^AA^ *p* < .01 vs. PBS, ^BB^ *p* < .01 vs. P2 MSC, ^CC^ *p* < .01 vs. P9 MSC, and ^DD^ *p* < .01 vs. P9 MSC + melatonin+siHSPA1L. (e) Engrafted cells were analyzed by immunofluorescence staining for human nuclear antigen (HNA; green). Scale bar = 50 μm. (f) The number of engrafted cells was quantified by counting the number of HNA‐positive cells. The values represent the means ± *SEM* (*n* = 3). ND, not detected, ^AA^ *p* < .01 vs. PBS, ^B^ *p* < .05 vs. P2 MSC, ^C^ *p* < .05 vs. P9 MSC, and ^D^ *p* < .05 vs. P9 MSC + melatonin+siHSPA1L

**Figure 6:**
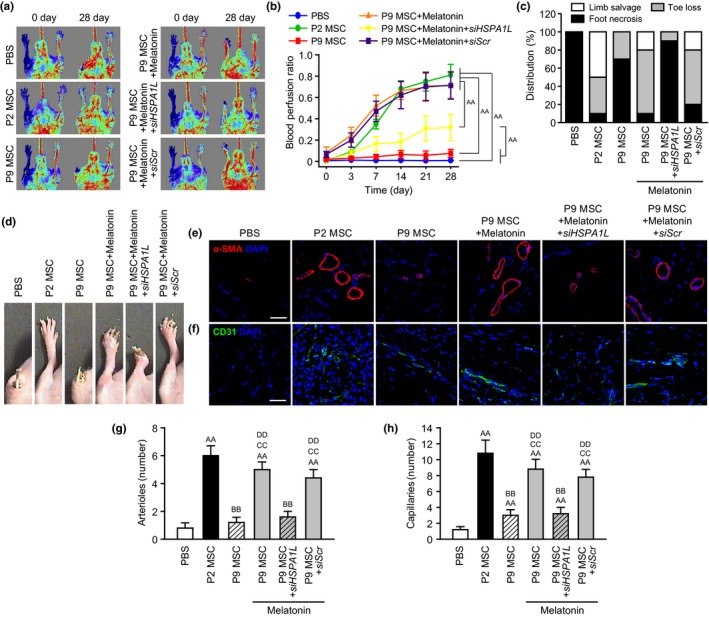
Assessment of functional recovery in a murine hindlimb ischemia model after transplantation of senescent MSCs. (a) Blood perfusion was assessed by laser Doppler perfusion imaging (LDPI) analysis. (b) Blood perfusion ratios (blood flow in the left ischemic limb/blood flow in the right nonischemic limb) were quantified by LDPI analysis. The values represent the means ± *SEM* (*n* = 10). ^AA^ *p* < .01. (c) Distribution of different outcomes (foot necrosis, toe loss, and limb salvage) in 28 days after the operation (*n* = 10). (d) Representative images illustrating various experimental outcomes in ischemic limbs in 28 days after the operation. (e and f) Arteriole and capillary formation were assessed by immunofluorescence staining for α‐SMA (e; red) and CD31 (f; green). Scale bar = 50 μm. (g and h) Standard quantification of the arteriole (g) and capillary (h) density represented as the number of α‐SMA‐ and CD31‐positive cells, respectively. The values represent the means ± *SEM* (*n* = 3). ^AA^ *p* < .01 vs. PBS, ^BB^ *p* < .01 vs. P2 MSC, ^CC^ *p* < .01 vs. P9 MSC, and ^DD^ *p* < .01 vs. P9 MSC + melatonin+siHSPA1L

Melatonin increased the expression of angiogenic cytokines, such as VEGF, FGF, and HGF, in senescent MSCs via HSPA1L expression (Figure S10). To assess the effect of melatonin‐treated senescent MSCs on functional recovery in ischemic tissues, we established a hindlimb ischemia model and assessed functional recovery and neovascularization after transplantation of melatonin‐treated senescent MSCs. At postoperative day 3, apoptosis and proliferation in ischemic tissues were analyzed following the injection with MSCs. Terminal deoxynucleotidyl transferase‐mediated dUTP nick end labeling (TUNEL) assay results indicated that the apoptotic cells in ischemic tissues injected with melatonin‐treated senescent MSCs (P9) were significantly reduced, compared to that in ischemic tissues injected with nontreated senescent MSCs (Figure 5a,b). In addition, immunofluorescence staining for proliferating cell nuclear antigen (PCNA) showed a significant increase in cell proliferation in tissues injected with melatonin‐treated senescent MSCs (Figure 5c,d). Furthermore, immunofluorescence staining for human nuclear antigen (HNA) indicated that melatonin significantly enhanced the engraftment of senescent MSCs in ischemic tissues (Figure 5e,f). However, these effects were abolished by knockdown of HSPA1L (Figure 5a–f). These results suggest that melatonin‐treated senescent MSCs improve the survival and proliferation in ischemic tissues via expression of HSPA1L. At postoperative day 28, the blood perfusion ratio and limb salvage were assessed to confirm the functional recovery in ischemic tissues. The blood perfusion ratio was significantly lower in the transplantation of senescent MSCs than that in the transplantation of healthy MSCs (P2), whereas transplantation of melatonin‐treated senescence MSCs exhibited significantly increased blood flow, compared to that in the transplantation of senescent MSCs (Figure S11, Figure 6a,b). Toe loss and foot necrosis were also significantly decreased in mice from the transplantation of melatonin‐treated senescent MSC group, compared to the extent of these pathologies in mice from the transplantation of senescent MSCs (Figure 6c,d). However, silencing of HSPA1L prevented the beneficial effects of melatonin‐treated MSCs on blood perfusion and limb salvage (Figure S11, Figure 6a–d). Capillary and arteriole densities were significantly increased in mice transplanted with melatonin‐treated senescence MSCs, compared to mice transplanted with nontreated senescent MSCs (Figure 6e–h). In contrast, knockdown of HSPA1L showed a significant reduction in neovascularization in melatonin‐treated senescent MSCs (Figure 6e–h). These results indicate that functional recovery and neovascularization in hindlimb ischemia are improved by enhancing survival and proliferation of melatonin‐treated senescent MSCs via HSPA1L.

## 3 DISCUSSION

A variety of studies have shown that melatonin protects against senescence associated with oxidative stress (Zhou et al., 2015), neurodegeneration (Caballero et al., 2008), and CKD (Han et al., 2019). Melatonin rescues oxidative stress‐induced premature senescence in MSCs through attenuation of p‐p38, inhibition of p16^INK4α^, and augmentation of SIRT1 (Zhou et al., 2015). It also prevents aberrant differentiation and senescence of MSCs from iron imbalance by inhibiting ROS accumulation and membrane potential depolarization through down‐regulation of p53, ERK, and p38 (Yang et al., 2017). Melatonin also suppresses CKD‐associated senescence in MSCs through upregulation of PrP^C^ (Han et al., 2019). We found that melatonin protects MSCs against replicative senescence‐mediated mitochondrial dysfunction through activation of mitophagy. Under physiological and pathophysiological conditions, the mechanisms for mitochondrial quality control are regulated by mitochondrial fission and fusion (Ni et al., 2015). Mitofission and fusion play important roles in cell growth, division, cellular distribution, and turnover of mitochondria (Mishra & Chan, 2014). To prevent the accumulation of damaged mitochondria by several stresses, dysfunctional mitochondria are divided into two heterogeneous sets of daughter mitochondria which have increased or decreased mitochondrial membrane potential. Daughter mitochondria with higher mitochondrial membrane potential, which is better quality mitochondria, proceed to mitofusion, whereas other depolarized mitochondria with lower mitochondrial membrane potential, which is characteristic of lower quality mitochondria, are degraded by mitophagy, resulting in a turnover of mitochondria (Twig et al., 2008). Our results show that replicative senescence in MSCs significantly increases the levels of mitofusion‐associated proteins, resulting in the inhibition of mitophagy. In contrast, treatment of senescent MSCs with melatonin removed impaired mitochondria by inhibiting mitofusion and proceeding to mitophagy. This study, for the first time, suggests that replicative senescence by ex vivo expansion of MSCs inhibits mitophagy of impaired mitochondria through the increase in mitofusion and melatonin rescues replicative senescence by activating mitophagy through mitochondrial quality control.

This study has verified that melatonin‐induced mitophagy in senescent MSCs is regulated by the recruitment of the HSPA1L‐PrP^C^ complex. HSPA1L contributes to protein stabilization, cell growth, apoptosis, and signal transduction (Hasson et al., 2013; Lee, Han, Yoon, et al., 2017; Lee et al., 2018; Mayer & Bukau, 2005; Wang et al., 2013). In damaged mitochondria of HeLa cells, HSPA1L and BAG family molecular chaperone regulator 4 induces the translocation of parkin to mitochondria, leading to mitochondrial quality control (Hasson et al., 2013). In colorectal cancer cells, HIF‐1α is stabilized by binding to HSPA1L (Lee, Han, Yoon, et al., 2017). Additionally, HSPA1L binds to OCT4 in cancer stem cells, resulting in maintenance of stemness (Lee et al., 2018). PrP^C^, the normal cellular prion protein, is a pivotal molecule with fundamental roles in self‐renewal, proliferation, and angiogenesis of stem/progenitor cells (Lee, Han, & Lee, 2017; Martin‐Lanneree et al., 2017; Martin‐Lanneree et al., 2014). Treatment with melatonin increases the function of MSCs through upregulation of PrP^C^, resulting in improvement of neovascularization in ischemic tissues (Lee, Han, & Lee, 2017). In CKD, melatonin‐induced PrP^C^ enhances mitochondrial function by binding to PINK1, leading to an increase in mitochondrial metabolism (Han et al., 2019). Our results show that melatonin‐induced PrP^C^ in senescent MSCs is regulated by binding to HSPA1L and that this complex facilitates the recruitment of PrP^C^ into mitochondria, resulting in reduced mitochondrial ROS production and increased oxidative phosphorylation. Furthermore, knockdown of HSPA1L inhibits the beneficial effect of melatonin in senescent MSCs. These findings suggest that melatonin increases the expression and the recruitment of PrP^C^ into mitochondria in senescent MSCs by binding to HSPA1L, indicating that the protective effects of melatonin against replicative senescence in MSCs are dependent on the HSPA1L expression.

To understand the precise mechanism by which the HSPA1L‐PrP^C^ complex regulates mitochondrial quality control in senescent MSCs, we revealed the interaction between PrP^C^ and COX4I1. COX4I1 is the principal isoform for cytochrome c oxidase (COX). COX is an enzyme of the mitochondrial respiratory chain which plays a key role in mitochondrial oxidative phosphorylation through a transfer of electrons from cytochrome c to oxygen, leading to the induction of the proton electrochemical gradient and mitochondrial membrane potential (Li, Park, Deng, & Bai, 2006). A clinical study has shown that the defective production of COX causes myopathy and Leigh syndrome (Pillai et al., 2019). Mutation in the human *COX4I1* gene induces short stature, poor weight gain, and chromosomal breaks (Abu‐Libdeh et al., 2017). Furthermore, low COX4I1 expression is associated with impaired ATP production and elevated ROS (Abu‐Libdeh et al., 2017). A recent study has indicated that a COX4I1‐HSP70 complex contributes to the formation of COX, leading to the maintenance of the mitochondrial membrane potential (Bottinger et al., 2013). Like this study, our results have shown that PrP^C^ binds to COX4IA and that this binding is inhibited by knockdown of HSPA1L, suggesting that a HSPA1L‐PrP^C^‐COX4I1 complex might be a key component for regulating mitochondrial quality control in senescent MSCs.

Although replicative senescence and stress‐induced premature senescence (SIPS), which is induced by various oxidative stresses including H_2_O_2_ and other chemicals inducing oxidative stress, share many cellular features including altered cell morphology, DNA damage, and inhibition of cell cycle (Ho et al., 2011; Moussavi‐Harami, Duwayri, Martin, Moussavi‐Harami, & Buckwalter, 2004; Yu et al., 2018), several studies found different phenotypes including the phase of cell cycle arrest, global DNA methylation, telomere length, protein profiles, and gene expression between replicative senescence and SIPS (Aan, Hairi, Makpol, Rahman, & Karsani, 2013; Bielak‐Zmijewska et al., 2014; Kural, Tandon, Skoblov, Kel‐Margoulis, & Baranova, 2016; Pascal et al., 2005). These findings suggest that it is important to select proper model for the study on senescence due to differences in the signal pathways and molecular mechanisms between replicative senescence and SIPS. Pathophysiological condition in several diseases reduces the therapeutic effect of MSC‐based therapy. Recent reviews have described preclinical studies in which melatonin improves MSC bioactivities, including the migration to injured sites, the increase in the anti‐oxidant effect, the augmentation of survival of transplanted MSCs, and alleviation of inflammation, in myocardial infarction, acute kidney disease, and limb ischemia (Hu & Li, 2019). Like these preclinical studies, we have shown that melatonin‐treated senescent MSCs enhance functional recovery in a murine hindlimb ischemia model by inhibiting apoptosis, increasing proliferation, and augmenting neovascularization via HSPA1L expression. These findings suggest that in order to expand human MSCs for preclinical and/or clinical applications, treatment with melatonin may be a powerful strategy for preventing replicative senescence. Taken together, this study reveals that melatonin protects human MSCs, which are expanded at the late passage, against replicative senescence through the recruitment of the HSPA1L‐PrP^C^‐COX41A complex into mitochondria, and the removal of dysfunctional mitochondria by mitophagy. We also found that melatonin‐induced HSPA1L is an important molecule for rescuing defective mitochondrial function. These findings suggest that the regulation of HSPA1L in MSCs might provide an important clue for preventing replicative senescence and regulating mitochondrial quality control.

## 4 EXPERIMENTAL PROCEDURES

All detailed experimental protocols and materials are presented in the Supporting Information 1.

### 4.1 Human MSCs cultures

This study was approved by the local ethic committee, and informed consent was obtained from all the study subjects. Human adipose tissue‐derived MSCs were obtained from Soonchunhyang University Seoul Hospital (Seoul, Republic of Korea; IRB: SCHUH 2017–10–016) according to a protocol approved by the Ehics Review Board of Soonchunhyang University Seoul Hospital. Cells were cultured in α‐minimum essential medium supplemented with 10% (v/v) of fetal bovine serum and 100 U/ml penicillin/streptomycin (Thermo Fisher Scientific). Melatonin (Sigma Aldrich) was dissolved in ethanol and stored at 4°C until further use.

### 4.2 Western blot analysis

The lysates from MSCs (passages 2 and 9) were separated by sodium dodecyl sulfate–polyacrylamide gel electrophoresis (SDS‐PAGE). The protein‐transferred membranes were incubated with the appropriate primary antibodies, followed by detection using HRP‐conjugated secondary antibodies (Cell Signaling Technology).

### 4.3 Flow cytometry analysis

The mitochondrial superoxide was measured using flow cytometry analysis for MitoSOX^TM^ (Thermo Fisher Scientific) and tetramethylrhodamine, ethyl ester (Abcam) staining.

### 4.4 Immunoprecipitation

The lysates were incubated with anti‐PrP^C^ antibody and then mixed with the Protein A/G PLUS‐Agarose Immunoprecipitation Reagent (Santa Cruz Biotechnology). The immunocomplexes were separated by SDS‐PAGE and assessed by Western blotting.

### 4.5 Superoxide dismutase activity

MnSOD activity was determined in MSCs using a SOD activity kit (Enzo Life Sciences).

### 4.6 Mitochondrial complex I and IV activity

Mitochondrial complex I and IV activities in MSCs were measured using a Complex I and IV Enzyme Activity Microplate Assay Kit (Abcam).

### 4.7 Senescence‐associated β‐galactosidase assay

Senescence‐associated β‐galactosidase activity was analyzed using a Senescence β‐Galactosidase Staining Kit (Cell Signaling Technology).

### 4.8 Ethics statement

All animal care procedures and experiments were approved by the Institutional Animal Care and Use Committee of Soonchunhyang University Seoul Hospital (IACUC2015‐5) and were performed in accordance with the National Research Council (NRC) Guidelines for the Care and Use of Laboratory Animals. The experiments were performed on 8‐week‐old male BALB/c nude mice (Biogenomics) maintained on a 12‐hr light/dark cycle at 25°C in accordance with the regulations of Soonchunhyang University Seoul Hospital.

### 4.9 Murine hindlimb ischemia model

Experiments using a murine hindlimb ischemia model were performed as previously reported with minor modifications (Limbourg et al., 2009). Blood perfusion was assessed using laser Doppler perfusion imaging (LDPI; Moor Instruments, Wilmington, DE, USA).

### 4.10 TUNEL assay

The TUNEL assay was performed using a TdT fluorescein in situ apoptosis detection kit (Trevigen Inc).

### 4.11 Immunofluorescence staining

The ischemic areas were isolated and embedded in paraffin. Immunofluorescence staining was performed using the appropriate primary antibodies followed by incubation with the secondary antibodies.

### 4.12 Statistical analysis

Data were expressed as the mean ± standard error of the mean (*SEM*). All experiments were evaluated using the one‐way analysis of variance (ANOVA). Comparisons of three or more groups were made using Tukey's post hoc test. A *p* value < .05 was considered statistically significant.

## CONFLICT OF INTEREST

The authors declare no conflict of interests.

## AUTHOR CONTRIBUTIONS

J.H.L. prepared the study concept and design, performed data acquisition, analysis interpretation, and drafting of the manuscript. Y.M.Y. and K‐H.S. performed data acquisition, analysis interpretation, and statistical analysis. H.N. provided cells. S.H.L. prepared the study concept and design, and performed data analysis and interpretation of data, and also drafting of the manuscript, procurement of funding, and study supervision.

## Data Availability

The data that support the findings of this study are available on request from the corresponding author. The data are not publicly available due to privacy or ethical restrictions. This study investigated mesenchymal stem cells which were isolated from adipose tissues of human individual (IRB: SCHUH 2017‐10‐016).
