# Supplementary material for: Melatonin suppresses senescence‐derived mitochondrial dysfunction in mesenchymal stem cells via the HSPA1L–mitophagy pathway
Source: Aging Cell. 2020 Jan 22;19(3):e13111. doi: 10.1111/acel.13111 (PMC7059143; doi:10.1111/acel.13111)

Supplemental Figure 1

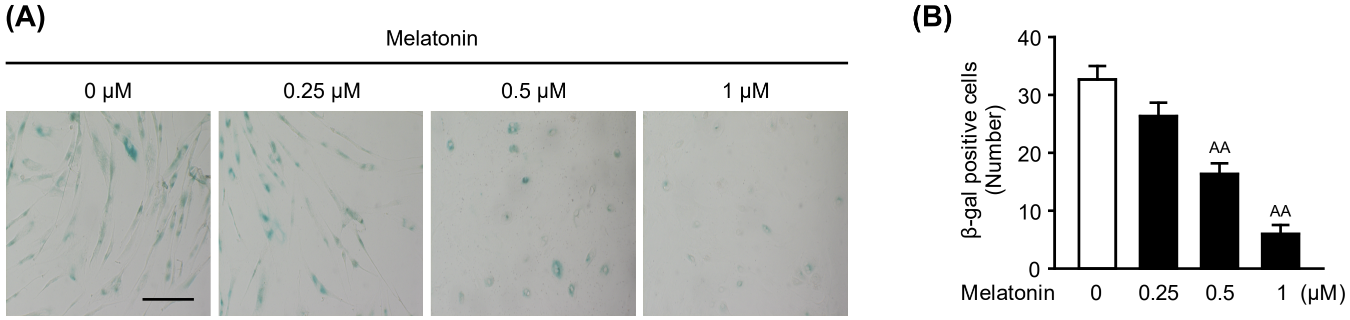

Supplemental Figure 2

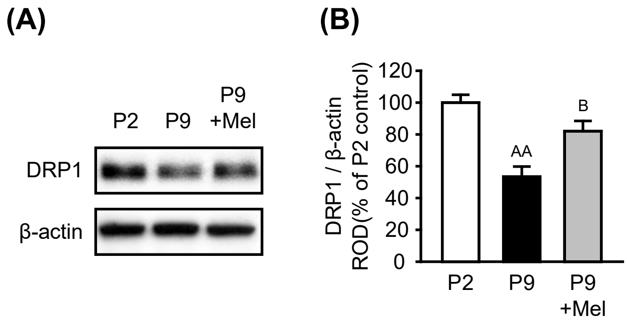

Supplemental Figure 3

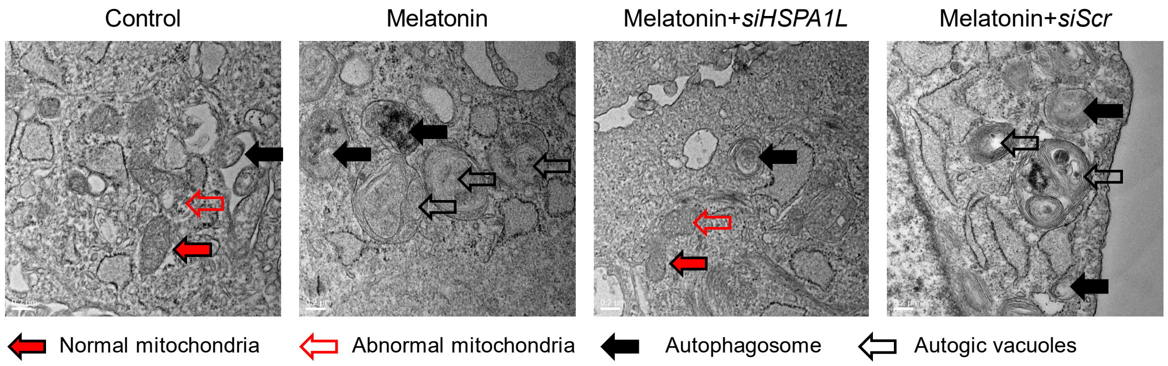

Supplemental Figure 4

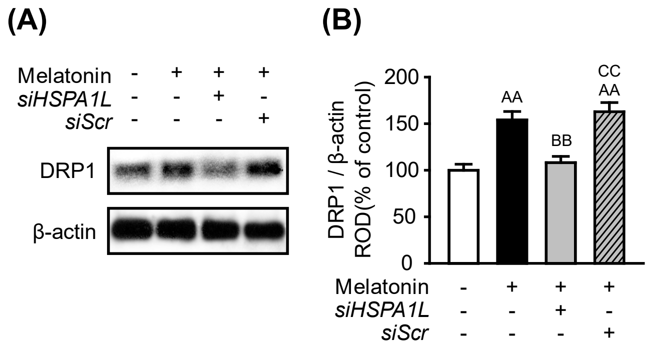

Supplemental Figure 5

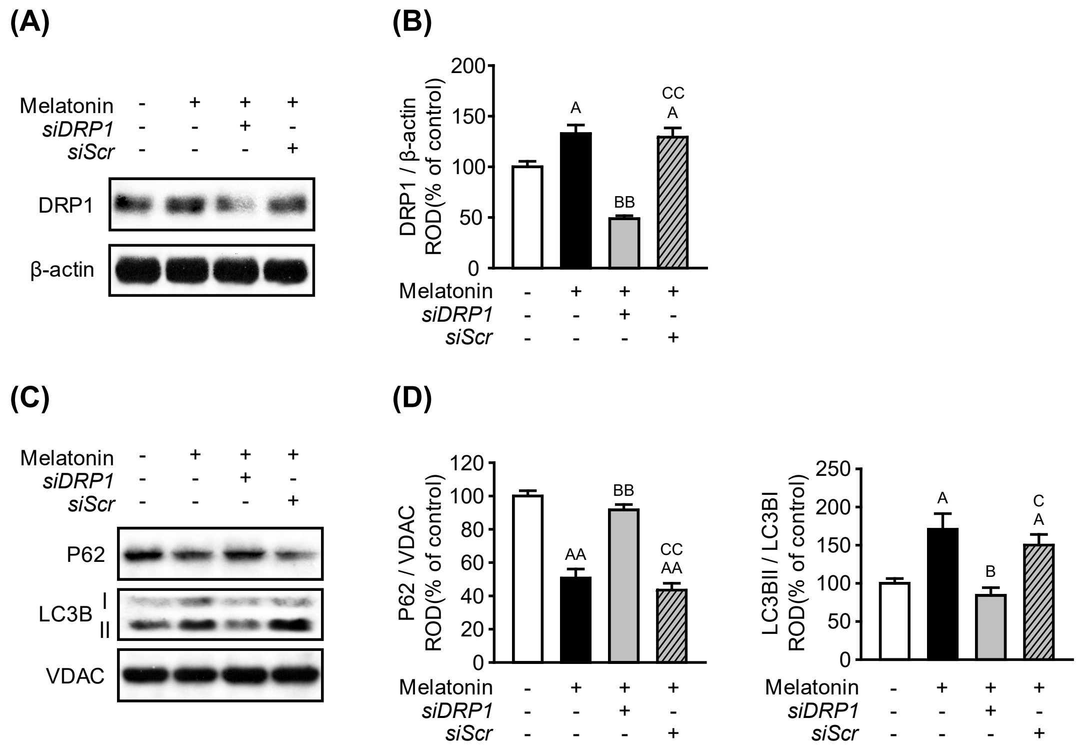

Supplemental Figure 6

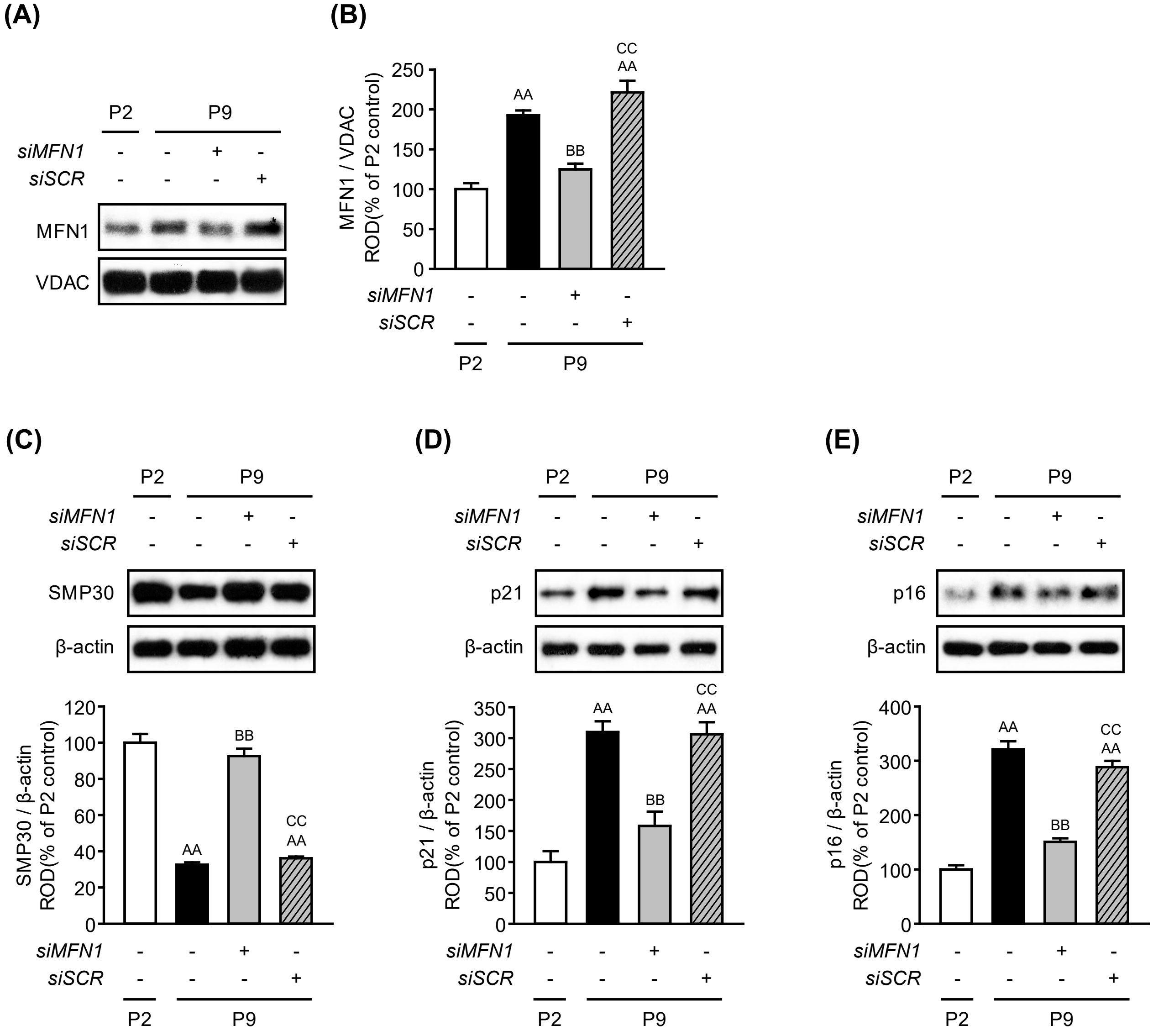

Supplemental Figure 7

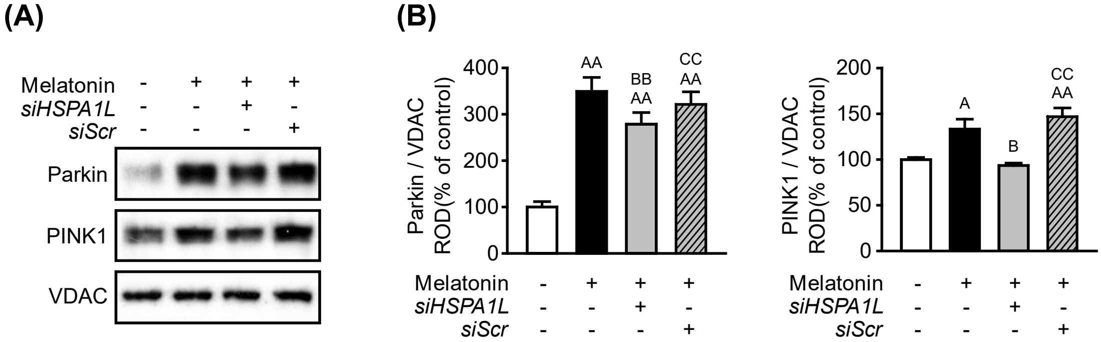

Supplemental Figure 8

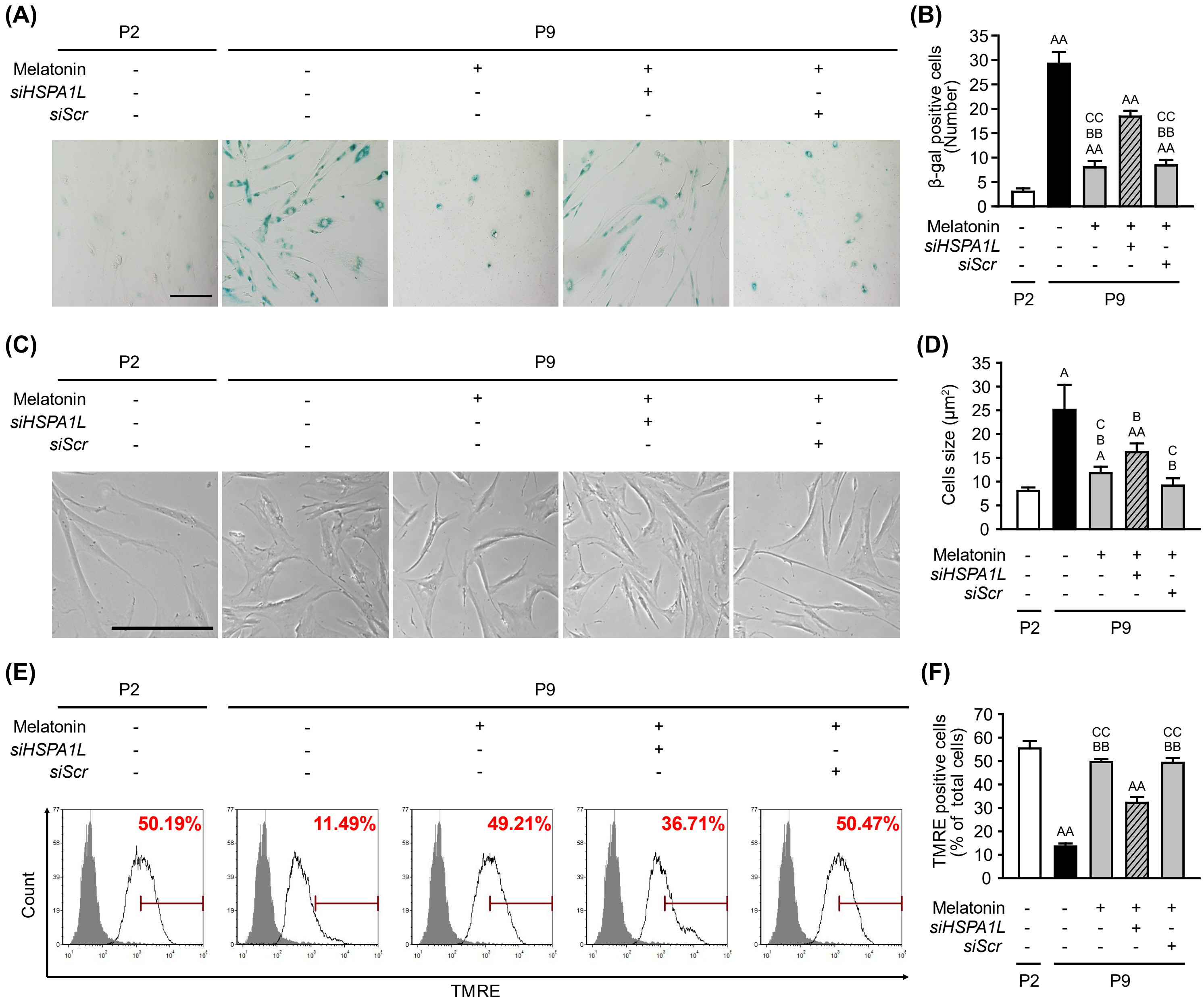

Supplemental Figure 9

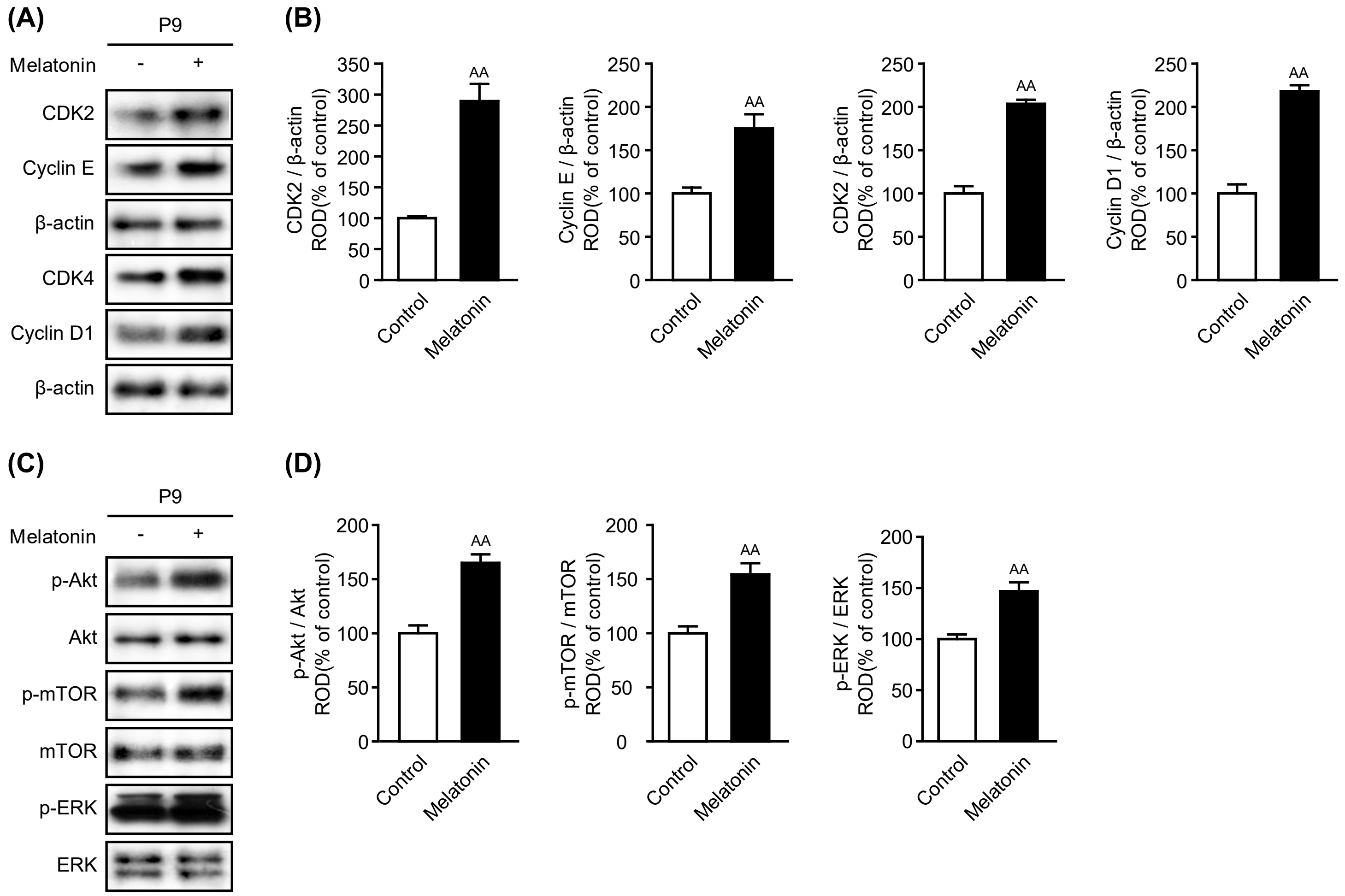

Supplemental Figure 10

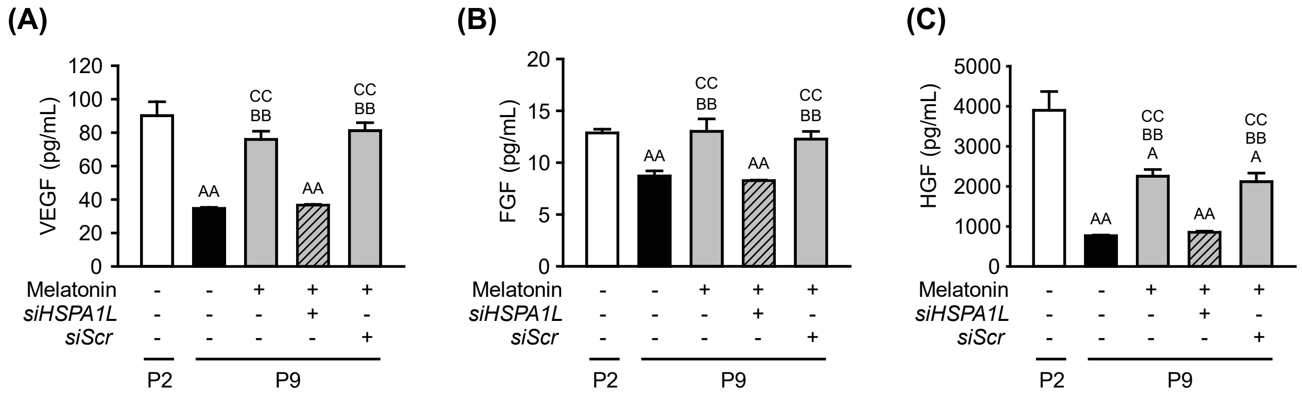

Supplemental Figure 11

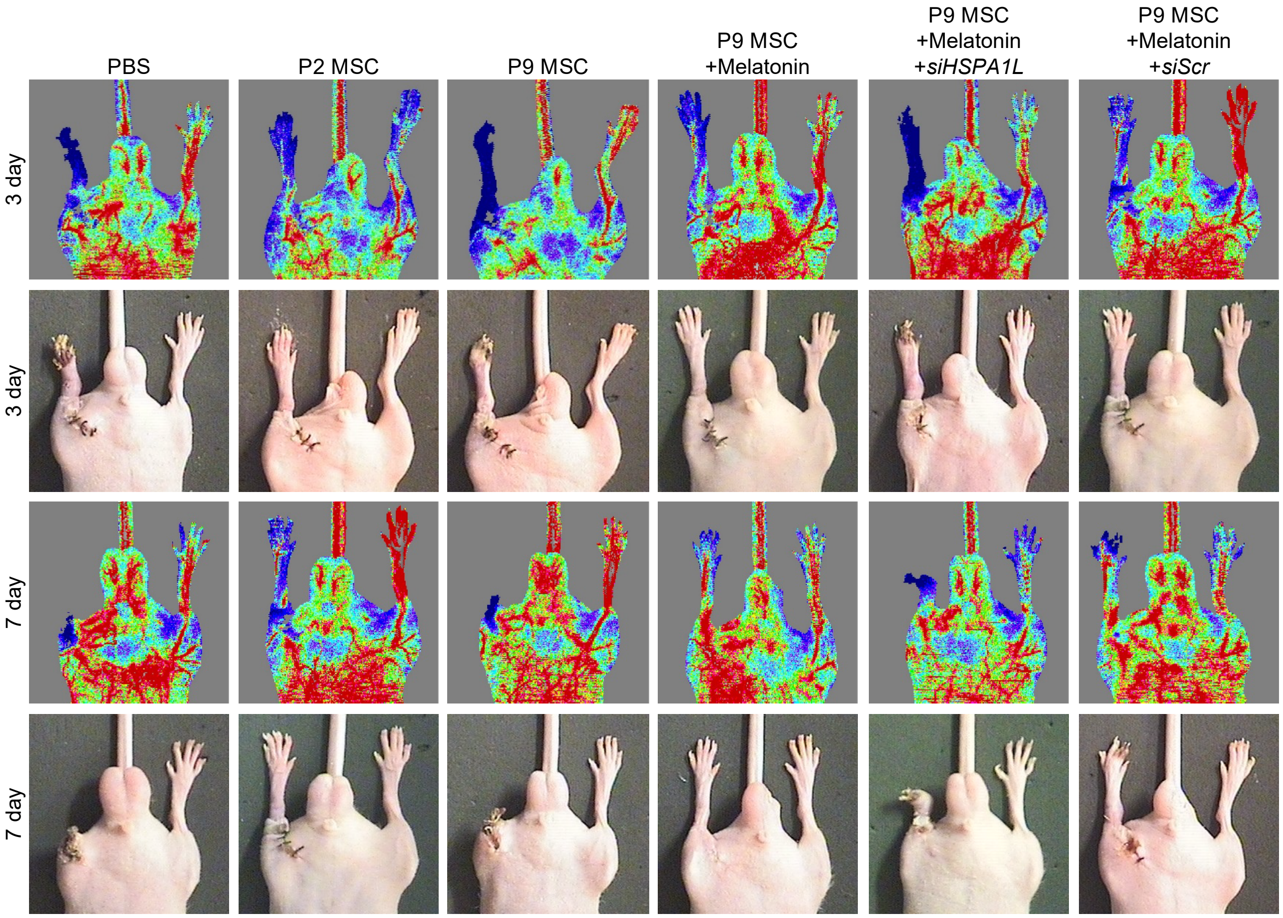

Supplement: Supplementary file 1 [file ACEL-19-e13111-s001.pdf]
