# Supplementary material for: Melatonin suppresses senescence‐derived mitochondrial dysfunction in mesenchymal stem cells via the HSPA1L–mitophagy pathway
Source: Aging Cell. 2020 Jan 22;19(3):e13111. doi: 10.1111/acel.13111 (PMC7059143; doi:10.1111/acel.13111)
Supplement: Supplementary file 2 [file ACEL-19-e13111-s002.docx]

**Supporting Information 1**

**1 | MATERIALS AND METHODS**

**1.1 | Human MSCs cultures**

This study was approved by the local ethic committee, and informed consent was obtained from all the study subjects. Human adipose tissue-derived MSCs were obtained from Soonchunhyang University Seoul Hospital (Seoul, Republic of Korea; IRB: SCHUH 2017-10-016) according to a protocol approved by the Ehics Review Board of Soonchunhyang University Seoul Hospital. The characterization of MSCs was assessed by the expression of MSC surface positive markers (CD44 and Sca-1) and negative markers (CD45 and CD11b) ([Yoon et al., 2019](#_ENREF_2)). MSCs were differentiated into adipogenic, chondrogenic, and osteogenic cells under conditions of specific differentiation media ([Yoon et al., 2019](#_ENREF_2)). Cells were cultured in α-minimum essential medium (α-MEM; Thermo Fisher Scientific, Waltham, MA, USA) supplemented with 10% (v/v) of fetal bovine serum (FBS; Thermo Fisher Scientific) and 100 U/ml penicillin/streptomycin (Thermo Fisher Scientific). To induce the replicative senescence in MSCs, cells were used at passage 9 in subsequent experiments. Cells were maintained in a humidified 5% CO_2_ incubator at 37°C. Melatonin (Sigma Aldrich, St. Louis, MO, USA) was dissolved in ethanol, filter-sterilized through a 0.45 μm pore filter (Sartorius, Göttingen, Germany), and stored as a 10 mM stock solution at 4°C until further use.

**1.2 | Electron microscopy**

MSCs were fixed in 3% glutaraldehyde and 2% paraformaldehyde in 100 mM sodium cacodylate buffer (pH 7.3). Morphometric analysis, including the number of mitochondria per cell and the size of mitochondria, was performed using ImageJ (NIH; version 1.43). To assess the number of mitochondria per cell, low-magnification images (× 10,000) were analyzed. To investigate the perimeter and the area, normal and abnormal mitochondria at high magnification (× 25,000) were assessed. Normal mitochondria have mostly longitudinally oriented and tightly packed cristae. Abnormal mitochondria have swollen, irregular, and whirling cristae.

**1.3 | Western blot analysis**

Whole cell proteins of MSCs were extracted using the RIPA lysis buffer (Thermo Fisher Scientific). Mitochondrial lysates from MSCs were isolated using Mitochondria Isolation Kit for Cultured Cells (Thermo Fisher Scientific) according to the manufacturer's instructions. The whole cell lysates and mitochondrial lysates from MSCs (passage 2 and 9) were separated by sodium dodecyl sulfate-polyacrylamide gel electrophoresis (SDS-PAGE). The proteins were transferred to a nitrocellulose membrane. The membranes were blocked with 5% (w/v) skimmed milk for 1 h at room temperature and then incubated with the appropriate primary antibodies against dynamin-1-like protein (DRP1; 1:1,000; cat. no. SC-271583; Santa Cruz Biotechnology, Dallas, TX, USA), phosphor-DRP1 at Ser637 (p-DRP1 (Ser637); 1:1,000; cat. no. 4867; Cell Signaling Technology, Danvers, MA, USA), mitofusin-1 (MFN1; 1:1,000; cat. no. SC-166644; Santa Cruz Biotechnology, Dallas, TX, USA), optic atrophy 1 (OPA1; 1:1,000; cat. no. NPB1-71656; Novus Biologicals, Centennial, CO, USA), HSPA1L (1:1,000; cat. no. NBP1-33022; Novus Biologicals), PrP^C^ (1:1,000; cat. no. SC-393165; Santa Cruz Biotechnology), voltage-dependent anion channels (VDAC; 1:1,000; cat. no. NB100-895; Novus Biologicals), cytochrome c oxidase subunit 4 isoform 1 (COX4I1; 1:1,000; cat. no. NB110-39115; Novus Biologicals), manganese superoxide dismutase (MnSOD; 1:1,000; cat. no. PA1-125; Thermo Fisher Scientific), p62 (1:1,000; cat. no. NBP1-45320; Novus Biologicals), microtubule-associated proteins 1A/1B light chain 3B (LC3B; 1:1,000; cat. no. NB100-2200; Novus Biologicals), senescence marker protein-30 (SMP30; 1:1,000; cat. no. SC-130344; Santa Cruz Biotechnology), p21 (1:1,000; cat. no. SC-51689; Santa Cruz Biotechnology), p16 (1:1,000; cat. no. 80772; Cell Signaling Technology), Parkin (1:1,000; cat. no. NBP2-29838; Novus Biologicals), PINK1 (1:1,000; cat. no. BC100-494; Novus Biologicals), cyclin-dependent kinase (CDK) 2 (1:1,000; cat. no. SC-6248; Santa Cruz Biotechnology), cyclin E (1:1,000; cat. no. SC-377100; Santa Cruz Biotechnology), CDK4 (1:1,000; cat. no. SC-56277; Santa Cruz Biotechnology), cyclin D1 (1:1,000; cat. no. SC-20044; Santa Cruz Biotechnology), protein kinase B (Akt; 1:1,000; cat. no. MAB2055; R&D systems, Minneapolis, MN, USA), p-Akt (1:1,000; cat. no. SC-101629; Santa Cruz Biotechnology), mammalian target of rapamycin (mTOR; 1:1,000; cat. no. NB100-240; Novus Biologicals), p-mTOR (1:1,000; cat. no. SC-101738; Santa Cruz Biotechnology), extracellular signal-regulated kinase (ERK; 1:1,000; cat. no. MAB1576; R&D systems), p-ERK (1:1,000; cat. no. SC-7383; Santa Cruz Biotechnology), and β-actin (1:3,000; cat. no. SC-47778; Santa Cruz Biotechnology). The primary antibodies were detected by means of HRP-conjugated secondary antibodies (Cell Signaling Technology). The bands were visualized by enhanced chemiluminescence (Sigma Aldrich).

**1.4 | Flow cytometry analysis**

To assess the formation of mitochondrial O_2_^•−^, the mitochondrial superoxide of MSCs was measured using flow cytometry analysis for MitoSOX^TM^ (Thermo Fisher Scientific) and Tetramethylrhodamine, ethyl ester (TMRE; Abcam, Cambridge, UK) staining. The cells were incubated with a 10 μM MitoSOX^TM^ solution or 200 nM TMRE solution in phosphate buffered saline (PBS) at 37°C for 15 min. After washing 2 times with PBS, cells were resuspended in 500 μL of PBS and analyzed using fluorescence-activated cell sorting (FACS; Sysmex, Kobe, Japan).

**1.5 | Immunoprecipitation**

The whole cell or mitochondrial fraction was lysed with lysis buffer (1% Triton X-100 in 50 mM Tris-HCl [pH 7.4] containing 150 mM NaCl, 5 mM EDTA, 2 mM Na_0_VO_4_, 2.5 mM Na_4_PO_7_, 100 mM NaF, and protease inhibitors). The lysates were incubated with anti-PrP^C^ antibody and then mixed with the Protein A/G PLUS-Agarose Immunoprecipitation Reagent (Santa Cruz Biotechnology) at 4°C for 4 h. The immunocomplexes were washed four times and separated by SDS-PAGE. The precipitated proteins were assessed by western blotting with the appropriate primary antibodies.

**1.6 | siRNA transfection**

In accordance with the manufacturer’s instructions, Lipofectamine 2000 reagent (Thermo Fisher Scientific) was used to transfect siRNAs into MSCs. More specifically, the cells were grown to 70% confluence in cell culture dishes, and then transfected for 48 h with SMART pool siRNAs specific to HSPA1L mRNA (Dharmacon, Lafayette, Co, USA).

**1.7 | Superoxide dismutase activity**

MnSOD activity was determined in MSCs using a SOD activity kit (Enzo Life Sciences, Farmingdale, NY, USA) according to the manufacturer's instructions. Briefly, the cells were lysed with 1× cell extraction buffer supplemented with protease inhibitors. Cell lysates were allowed to react with SOD, the signals were immediately measured every minute by a microplate reader (BMG Labtech, Ortenberg, Germany) at the optical density 450 nm for 10 min.

**1.8 | Mitochondrial complex I and IV activity**

Mitochondrial complex I and IV activities in MSCs were measured using a Complex I and IV Enzyme Activity Microplate Assay Kit (Abcam), following the manufacturer’s instructions. Briefly, the submitochondrial fractions (0.6 mg/mL) were incubated for 3 minutes in a mitochondrial complex assay medium (250 mM sucrose, 50 mM potassium phosphate, 1 mM KCN, 50 μM decylubiquinone, and 0.8 μM antimycin, pH 7.4). Mitochondrial complex I activity was assessed from the rate of the oxidation of NADH (100 mM) by a microplate reader (BMG Labtech) at the optical density 340 nm. Mitochondrial complex IV activity was analyzed by adding cytochrome c, previously reduced with sodium borohydride, and determining the absorbance at 550 nm.

**1.9 | Senescence-associated β-galactosidase assay**

Senescence-associated β-galactosidase activity in MSCs was analyzed using a Senescence β-Galactosidase Staining Kit (Cell Signaling Technology) according to the manufacturer’s protocols. To ensure consistency in the cell density, 2,000 cells were seeded on each well in a 96 well plates. After staining, images were obtained using a light microscope (Nikon, Tokyo, Japan).

**1.10 | Ethics statement**

All animal care procedures and experiments were approved by the Institutional Animal Care and Use Committee of Soonchunhyaning University Seoul Hospital (IACUC2015-5) and were performed in accordance with the National Research Council (NRC) Guidelines for the Care and Use of Laboratory Animals. The experiments were performed on 8-week-male BALB/c nude mice (Biogenomics, Seoul, Korea) maintained on a 12 h light/dark cycle at 25°C in accordance with the regulations of Soonchunhyang University Seoul Hospital.

**1.11 | Murine hindlimb ischemia model**

Experiments using a murine hindlimb ischemia model were performed as previously reported with minor modifications ([Limbourg et al., 2009](#_ENREF_1)). Briefly, ischemia was induced in mice by the ligation and excision of the proximal femoral artery and boundary vessels. No later than 6 h after surgery, mice were injected intramuscularly in the ischemic thigh area with one of the following: PBS, P2 MSCs (early passage), P9 MSCs (late passage; senescent cells), melatonin-treated P9 MSCs, melatonin-treated P9 MSCs pretreated with *siHSPA1L*, or melatonin-treated P9 MSCs pretreated with *siSCR* (5×10^5^ cells/100 μL PBS per mouse; 10 mice per group). Each mouse was given injections of cells into five ischemic sites. Blood perfusion was assessed by measuring the ratio of the blood flow in the ischemic (left) limb to that in the nonischemic (right) limb on postoperative days 0, 3, 7, 14, 21, and 28 using laser Doppler perfusion imaging (LDPI; Moor Instruments, Wilmington, DE, USA).

**1.12 | The terminal deoxynucleotidyl transferase-mediated dUTP nick end labeling (TUNEL) assay**

The TUNEL assay was performed using a TdT fluorescein *in situ* apoptosis detection kit (Trevigen Inc, Gaithersburg, MD, USA). At postoperative day 3, the TUNEL assay was performed in the ischemic injured tissues. Stained sections were observed using a confocal microscope (Olympus, Tokyo, Japan)

**1.13 | Immunofluorescence staining**

After 3 and 28 days following the surgery, the ischemic injured thigh areas were isolated and fixed with 4% paraformaldehyde. Each tissue sample was embedded in paraffin. Immunofluorescence staining was performed using primary antibodies against proliferating cell nuclear antigen (PCNA; Santa Cruz Biotechnology), CD31 (Santa Cruz Biotechnology), and α-smooth muscle actin (SMA; Santa Cruz Biotechnology) followed by incubation with the secondary antibodies conjugated to Alexa488 and Alexa594 (Thermo Fisher Scientific). Nuclei were stained with 4′,6-diamidine-2′-phenylindole dihydrochloride (Sigma Aldrich). Stained sections were visualized by confocal microscopy (Olympus).

**1.14 | Detection of human angiogenic cytokines**

Expression of VEGF, FGF, and HGF in MSCs were determined with commercially available ELISA kits (R&D systems). Expression levels of angiogenic cytokines were quantified by measuring absorbance at 450 nm on the microplate reader (BMG Labtech).

**1.15 | Statistical analysis**

Data were expressed as the mean ± standard error of the mean (SEM). All experiments were evaluated using the one-way analysis of variance (ANOVA). Comparisons of three or more groups were made using Tukey's post-hoc test. A *p* value < 0.05 was considered statistically significant.

**References**

Limbourg, A., Korff, T., Napp, L. C., Schaper, W., Drexler, H., & Limbourg, F. P. (2009). Evaluation of postnatal arteriogenesis and angiogenesis in a mouse model of hind-limb ischemia. *Nat Protoc, 4*(12), 1737-1746. doi: 10.1038/nprot.2009.185

Yoon, Y. M., Kim, S., Han, Y. S., Yun, C. W., Lee, J. H., Noh, H., & Lee, S. H. (2019). TUDCA-treated chronic kidney disease-derived hMSCs improve therapeutic efficacy in ischemic disease via PrP(C). *Redox Biol, 22*, 101144. doi: 10.1016/j.redox.2019.101144
