# Supplementary material for: Melatonin suppresses senescence‐derived mitochondrial dysfunction in mesenchymal stem cells via the HSPA1L–mitophagy pathway
Source: Aging Cell. 2020 Jan 22;19(3):e13111. doi: 10.1111/acel.13111 (PMC7059143; doi:10.1111/acel.13111)
Supplement: Supplementary file 3 [file ACEL-19-e13111-s003.docx]

**Supporting Information 2**

Supplemental Figure Legend

**Figure S1** Melatonin protects against replicative senescence in MSCs.

(A) Senescence-associated β-galactosidase (SA-β-gal) assay in senescent MSCs (P9) after treatment with melatonin (0, 0.25, 0.5, and 1 μM). Scale bar = 100 μm. (B) Number of SA-β-galactosidase-positive cells. The values represent the means ± SEM (n = 3). ^AA^*p* < 0.01 vs senescent MSCs (P9).

**Figure S2** Expression of total DRP1 in senescent MSCs treated with melatonin.

(A) Expression of total DRP1 in healthy MSCs (P2), senescent MSCs (P9), and senescent MSCs treated with melatonin (P9 + Mel). (B) The level of total DRP1 was determined by densitometry relative of β-actin. Values represent the mean ± SEM (n = 3). ^AA^*p* < 0.01 vs P2 and ^B^*p* < 0.05 vs P9.

**Figure S3** Melatonin facilitates the formation of autophagosome via expression of HSPA1L.

Representative TEM images of senescent MSCs (P9) after treatment with melatonin.

**Figure S4** Melatonin regulates the expression of DRP1 in senescent MSCs via HSPA1L expression.

(A) Expression of total DRP1 in senescent MSCs. (B) The level of total DRP1 was determined by densitometry relative of β-actin. Values represent the mean ± SEM (n = 3). ^AA^*p* < 0.01 vs non-treated senescent MSCs, ^BB^*p* < 0.01 vs melatonin-treated senescent MSCs, and ^CC^*p* < 0.01 vs melatonin-treated senescent MSCs pretreated with *siHSPA1L*.

**Figure S5** Melatonin increases mitophagy in senescent MSCs by regulating mitofusion.

(A) Expression of DRP1 in senescent MSCs pretreated with *DRP1* siRNA (*siDRP1*). (B) The level of DRP1 was determined by densitometry relative of β-actin. Values represent the mean ± SEM (n = 3). ^A^*p* < 0.05 vs non-treated senescent MSCs, ^BB^*p* < 0.01 vs melatonin-treated senescent MSCs, and ^CC^*p* < 0.01 vs melatonin-treated senescent MSCs pretreated with *siDRP1*. (C) Expression of p62 and LC3B in mitochondrial fraction of melatonin-treated senescent MSCs pretreated with *siDRP1*. (D) The levels of p62 and LC3BII/LC3BI were determined by densitometry relative of VDAC. Values represent the mean ± SEM (n = 3). ^A^*p* < 0.05; ^AA^*p* < 0.01 vs non-treated senescent MSCs, ^B^*p* < 0.05; ^BB^*p* < 0.01 vs melatonin-treated senescent MSCs, and ^C^*p* < 0.05; ^CC^*p* < 0.01 vs melatonin-treated senescent MSCs pretreated with *siDRP1*.

**Figure S6** MNF1 is involved in replicative senescence in MSCs.

(A) Expression of MFN1 in the mitochondria fraction of MSCs pretreated with *MFN1* siRNA (*siMFN1*). (B) The level of MFN1 was determined by densitometry relative of VDAC. Values represent the mean ± SEM (n = 3). ^AA^*p* < 0.01 vs P2 MSCs, ^BB^*p* < 0.01 vs P9 MSCs, and ^CC^*p* < 0.01 vs P9 MSCs pretreated with *siMFN1*. (C-E) Expression of SMP 30 (C), p21 (D), and p16 (E) in senescent MSCs pretreated with *siMFN1*. The levels of SMP 30 (C), p21 (D), and p16 (E) were determined by densitometry relative of β-actin. Values represent the mean ± SEM (n = 3). ^AA^*p* < 0.01 vs P2 MSCs, ^BB^*p* < 0.01 vs P9 MSCs, and ^CC^*p* < 0.01 vs P9 MSCs pretreated with *siMFN1*.

**Figure S7** Melatonin regulates expression of Parkin and PINK1 in mitochondira through HSPA1L level.

(A) Expression of Parkin and PINK1 in mitochondrial fraction of senescent MSCs. (B) The levels of Parkin and PINK1 were determined by densitometry relative of VDAC. Values represent the mean ± SEM (n = 3). ^A^*p* < 0.05; ^AA^*p* < 0.01 vs non-treated senescent MSCs, ^B^*p* < 0.05; ^BB^*p* < 0.01 vs melatonin-treated senescent MSCs, and ^CC^*p* < 0.01 vs melatonin-treated senescent MSCs pretreated with *siHSPA1L*.

**Figure S8** Treatment with melatonin inhibits replicative senescence of MSCs.

(A) Senescence-associated β-galactosidase (SA-β-gal) assay in healthy MSCs (P2) and senescent MSCs (P9), which are isolated from different donor compared to cells in main manuscript, after treatment with melatonin. Scale bar = 100 μm. (B) Number of SA-β-galactosidase-positive cells. The values represent the means ± SEM (n = 5). ^AA^*p* < 0.01 vs healthy MSCs (P2), ^BB^*p* < 0.01 vs senescent MSCs (P9), and ^CC^*p* < 0.01 vs melatonin-treated senescent MSCs (P9) pretreated with *siHSPA1L*. (C) Morphology of healthy MSCs (P2) and senescent MSCs (P9) after treatment with melatonin. Scale bar = 250 μm. (D) Quantification of cell size. The values represent the means ± SEM (n = 5). ^A^*p* < 0.05; ^AA^*p* < 0.01 vs healthy MSCs (P2), ^B^*p* < 0.05 vs senescent MSCs (P9), and ^C^*p* < 0.05 vs melatonin-treated senescent MSCs (P9) pretreated with *siHSPA1L*. (E) Flow cytometry for TMRE in healthy MSCs (P2) and senescent MSCs (P9) after treatment with melatonin. (F) Quantification of TMRE positive cells. Values represent the mean ± SEM (n = 3). ^AA^*p* < 0.01 vs healthy MSCs (P2), ^BB^*p* < 0.01 vs senescent MSCs (P9), and ^CC^*p* < 0.01 vs melatonin-treated senescent MSCs (P9) pretreated with *siHSPA1L*.

**Figure S9** Effect of melatonin on cell proliferation-associated signal pathway in senescent MSCs

(A) Expression of CDK2, cyclin E, CDK4, and cyclin D1 in senescent MSCs (P9) after treatment with melatonin. (B) The levels of CDK2, cyclin E, CDK4, and cyclin D1 were determined by densitometry relative of β-actin, respectively. Values represent the mean ± SEM (n = 3). ^AA^*p* < 0.01. (C) Expression of p-Akt, p-mTOR, and p-ERK in senescent MSCs (P9) after treatment with melatonin. (D) The levels of p-Akt, p-mTOR, and p-ERK were determined by densitometry relative of Akt, mTOR, and ERK, respectively. Values represent the mean ± SEM (n = 3). ^AA^*p* < 0.01.

**Figure S10** Melatonin increases secretion of angiogenic cytokines in senescent MSCs through upregulation of HSPA1L.

(A-C) The expression of angiogenic cytokines, including VEGF (A), FGF (B), and HGF (C), in senescent MSCs after treatment with melatonin. The levels of angiogenic cytokines were assessed by ELISA. Values represent the mean ± SEM (n = 5). ^A^*p* < 0.05; ^AA^*p* < 0.01 vs healthy MSCs (P2), ^BB^*p* < 0.01 vs senescent MSCs (P9), and ^CC^*p* < 0.01 vs melatonin-treated senescent MSCs (P9) pretreated with *siHSPA1L*.

**Figure S11** Assessment of functional recovery in a murine hindlimb ischemia model after transplantation of senescent MSCs at earlier stages.

Blood perfusion was assessed by laser Doppler perfusion imaging (LDPI) analysis at postoperative day 3 and 7.
